# Growth rates in non-syndromic aneurysms of the ascending aorta: a systematic review

**DOI:** 10.1007/s12471-024-01911-6

**Published:** 2024-11-19

**Authors:** Luc Cozijnsen, Bouke P. Adriaans, Tjard R. Schermer, Maarten Groenink, Simon Schalla, Sebastiaan C. A. M. Bekkers

**Affiliations:** 1 Department of Cardiology, Gelre Hospital, Apeldoorn, The Netherlands; 2 Cardiovascular Research Institute Maastricht (CARIM), Maastricht University, Maastricht, The Netherlands; 3 Department of Radiology and Nuclear Medicine, Maastricht University Medical Centre, Maastricht, The Netherlands; 4 Department of Cardiology, Maastricht University Medical Centre, Maastricht, The Netherlands; 5 Department of Clinical Epidemiology and Statistics, Gelre Hospital, Apeldoorn, The Netherlands; 6 Department of Cardiology, Amsterdam University Medical Centre, Amsterdam, The Netherlands; 7 Department of Cardiology, Bernhoven Hospital, Uden, The Netherlands

**Keywords:** Ascending aortic aneurysm, Thoracic aortic aneurysm, Dilatation rate, Growth rate

## Abstract

The maximum aortic diameter is the main risk predictor for type A acute aortic syndromes and understanding the growth rate of ascending thoracic aortic aneurysms (aTAAs) is pivotal for risk assessment and stratification for pre-emptive aortic surgery. Prevailing guidelines recommend serial imaging of aTAA patients until the thresholds for prophylactic surgery are met. Based on early landmark studies, it was thought that the growth rate of aTAAs is substantially higher than that of the normal-sized aorta. However, more recent studies have reported that aTAA growth is generally slow, questioning the need for frequent imaging during follow-up. The current systematic review provides an overview of studies reporting annual diameter growth rates of non-syndromic aTAAs and explains differences in findings between early and recent studies.

**Supplementary Information:**

The online version of this article (10.1007/s12471-024-01911-6) contains supplementary material, which is available to authorized users.

## Introduction

Aneurysms of the thoracic aortic may grow asymptomatically until they dissect or rupture with subsequent high mortality. The pooled incidence of thoracic aortic aneurysms and ruptured aneurysms was recently estimated to be 5.3 and 1.6 per 100,000 individuals per year, respectively [1]. Acute aortic syndromes (AAS) such as rupture and dissection occur with increasing aortic diameters [2–5]. To prevent AAS, pre-emptive surgery for aneurysms of the ascending thoracic aorta (aTAAs) is generally recommended for diameters ≥ 55 mm in non-syndromic patients [4, 6, 7], while some studies suggest prophylactic intervention in cases of smaller cross-sectional dimensions [8].

However, the prediction of AAS and stratification of patients for preventive surgery remains challenging. Understanding the natural history and growth rate of aTAAs is pivotal in assessing the risk of AAS [9]. The non-dilated ascending aorta expands with age. During middle to late adulthood, it increases by 0.7–0.9 mm/decade [10], with the maximum diameter usually not exceeding 40 mm [7]. Three early landmark studies reported higher growth rates (0.7–1.4 mm/year) once the aorta becomes dilated [2, 3, 11]. Based on these studies, the typical mean growth rate of aTAAs was assumed to be approximately 1.0 mm/year [5, 12, 13].

**Fig. 1:**
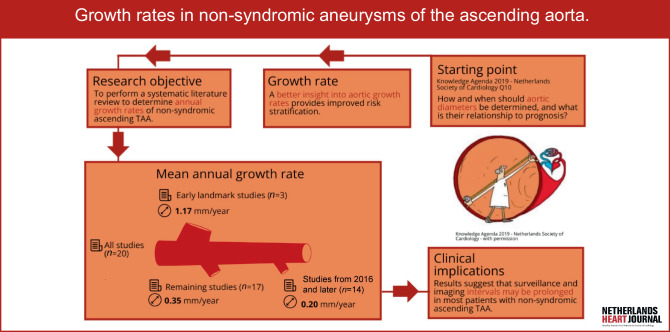
Infographic illustrating the starting point of the literature study, process and end results

Guideline recommendations for follow-up of aTAA patients have been based on this estimated diameter growth rate, and annual imaging has been recommended in aTAA patients who do not yet meet the indication criteria for pre-emptive surgery [6, 7, 14, 15]. However, multiple recent studies have demonstrated that aTAA growth rates are substantially lower than initially reported, suggesting that surveillance intervals might be prolonged [16, 17]. The Knowledge Agenda 2019 of the Netherlands Society of Cardiology defined this as a knowledge gap, which was the starting point of our study (Infographic, Fig. 1).

The aims of this literature review were: (1) to provide an overview of studies reporting annual diameter growth rates of non-syndromic aTAAs (which will frequently be sporadic or coincidently found aTAAs or aortopathy associated with bicuspid aortic valve (BAV)), and (2) to compare the reported growth rates from the three early landmark studies with those observed more recently. In addition, annual growth rates in aTAAs associated with BAV were compared to those in patients with tricuspid aortic valves (TAVs).

## Methods

We report on this systematic review in accordance with the Preferred Reporting Items for Systematic Reviews and Meta-analyses (PRISMA) reporting guideline. This study was not preregistered.

### Data sources and searches

A MEDLINE/PubMed search for English-language publications up to December 2023 was performed by a certified librarian, using the following Medical Subject Headings (MeSH) and search terms: ‘thoracic aortic aneurysm’, ‘ascending aortic aneurysm’, ‘aortic dissection’, ‘aortic root’ AND ‘growth rate’, ‘dilatation rate’, ‘progression rate’, ‘moderately dilated’ or ‘natural history’ (see Electronic Supplementary Material for the complete list of search terms). The reference lists of all selected articles were screened for additional potentially eligible studies.

### Eligibility criteria

Studies were included if: (1) they were original research studies in adult patients with non-syndromic aTAAs, (2) they were conducted in non-operated patients without prior aortic dissection, and (3) mean growth rates in millimetres per year were reported or could be retrieved. Studies that included patients with syndromic aTAAs (such as Marfan syndrome (MFS), Ehlers Danlos syndrome, Loeys-Dietz syndrome), or patients with complex congenital heart disease were excluded. Studies were not excluded on the basis of native aortic valve anatomy. As a result, the studies included comprised a mixture of patients with TAV and/or BAV, allowing comparison between the two valve types. Of note is that the three early landmark studies [2, 3, 11] did not fulfil our predefined eligibility criteria but were nonetheless included, because they form the basis for prevailing guidelines and allowed comparison with growth rates observed in recent studies.

### Data synthesis and analysis

After screening of titles and abstracts by one reviewer (LC), the full texts of selected publications were assessed by two reviewers (LC, BPA), followed by extraction of relevant data. Mean aortic growth rates were extracted, measured at the site of their largest diameter (either root or tubular segment). Study details were presented in tables. Growth rates and 95% confidence intervals (95% CIs) of the individual studies as well as the pooled overall weighted mean were visualised as forest plots (primary outcome of this review). In these forest plots mean aortic growth rates were sorted chronologically. Subsequently the plots were visually inspected to assess whether there were any noticeable differences over time.

The reported mean growth rates from three early landmark studies were compared with those observed more recently (secondary outcome). In addition, mean growth rates between patients with TAV and BAV were compared (additional aim). To statistically test differences between two overall weighted mean growth rates, meta-regression analysis (using a random effect model) was applied (IBM SPSS Statistics for Windows, Version 28.0, IBM Corp. Released 2021. IBM Corp., Armonk, NY). A two-sided *p*-value < 0.05 was considered statistically significant.

To assess confidence in the body of evidence, we used the Oxford Centre for Evidence-Based Medicine (OCEBM) classification (https://www.cebm.ox.ac.uk/resources/levels-of-evidence/ocebm-levels-of-evidence).

## Results

### Selection of studies

**Fig. 2:**
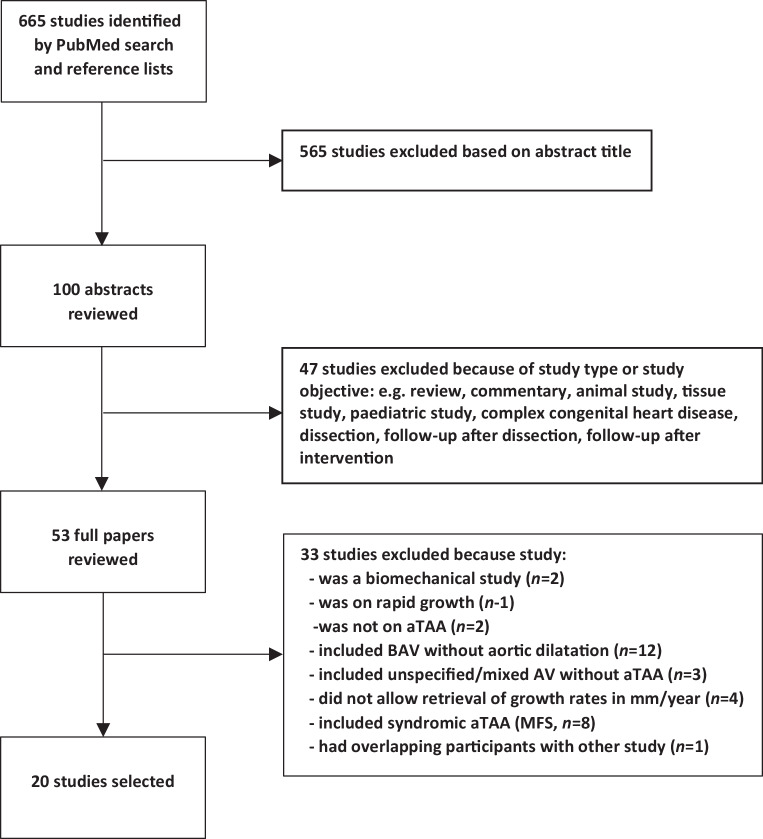
Flowchart showing selection of studies. Title and abstract screening was performed by one reviewer (LC) and full text screening by two reviewers (LC and BPA). *aTAA* ascending thoracic aortic aneurysm, *AV* aortic valve, *BAV* bicuspid aortic valve, *MFS* Marfan syndrome

**Fig. 3:**
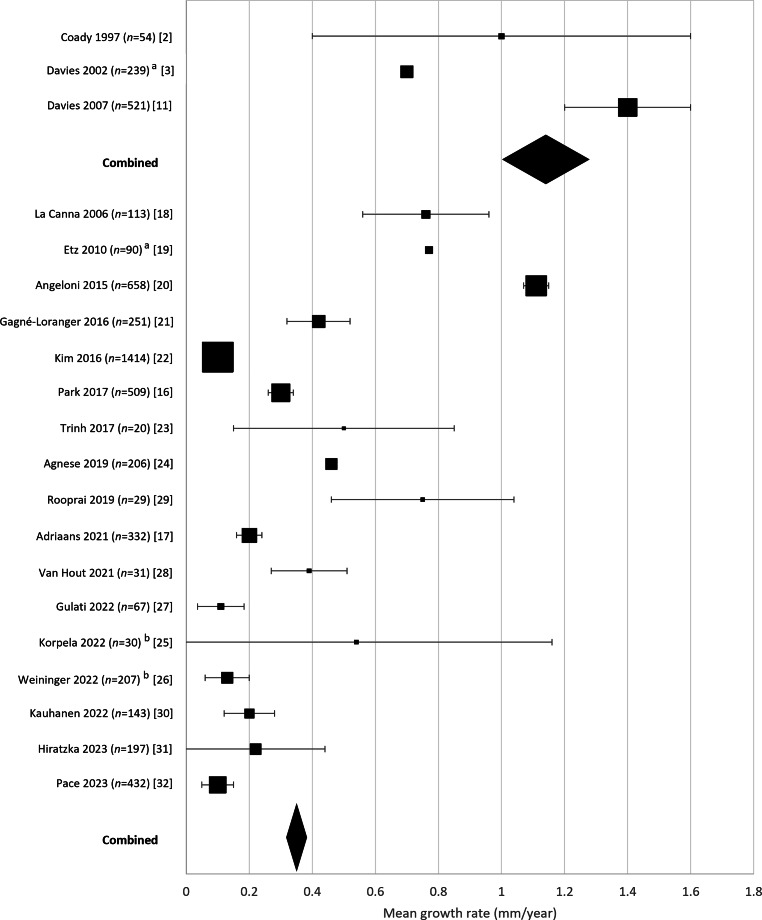
Growth rate from 20 ascending thoracic aortic aneurysm studies with unspecified or mixed aortic valve, tricuspid aortic valve or bicuspid aortic valve. *Square size* indicates sample size, *error bars* indicate 95% confidence intervals (95% CIs). *Diamonds *indicate the weighted mean growth rate of the combined studies and their 95% CIs. The *upper part* shows three early landmark studies (pooled mean 1.17 mm/year, 95% CI 1.01–1.33; [2, 3, 11]) The *lower part* shows the remaining 17 more recent studies (pooled mean 0.35 mm/year, 95% CI 0.33–0.37). The pooled mean of all 20 studies was 0.47 mm/year (95% CI 0.44–0.50). ^a^95% confidence interval could not be calculated based on the results as reported by the authors [3, 19]. ^b^ Mean and standard deviation estimated from the median and interquartile range as reported by the authors [25, 26]

The search strategy yielded a total of 665 studies, of which 565 were excluded after screening of titles. After reviewing the abstracts of the remaining 100 studies, 47 studies were additionally excluded because they were not original research articles or included non-adult patients, patients with complex congenital heart disease, or patients with previous AAS or aortic (valve) surgery. After reviewing the full text of the remaining 53 studies, 33 studies were excluded because they were biomechanical studies (*n* = 2), they included patients with non-dilated ascending aortas (*n* = 17), annual growth rates could not be retrieved (*n* = 4), patients with syndromic aTAAs were included (*n* = 8), only rapid growth rate was studied (*n* = 1) or participants overlapped with those in another study (*n* = 1). Finally, 20 studies were left for inclusion in this review (Fig. 2; [2, 3, 11, 16–32]). All of the 20 studies included were conducted in a single centre, and only three were designed prospectively [18, 21, 25].

### Mean annual growth rates from studies over time

Detailed information on the studies included is presented in Tab. 1 and 2. Mean annual growth rates are displayed as forest plots in Fig. 3. The pooled mean growth rate of the three early studies referred to in guidelines [2, 3, 11] was 1.17 mm/year (95% CI 1.01–1.33; *n* = 814). In contrast, the pooled mean growth rate of the remaining 17 more recent publications was 0.35 mm/year (95% CI 0.33–0.37; *n* = 4729). Meta-regression analysis showed a statistically significant difference in mean annual growth rate between these two subsets of studies (*p* = 0.008).

**Table 1:** Studies published before 2016

Study	Objective, mm	Period	Participants	Mean age, years	Male,%	Morphology/therapy	Imaging	Mean follow-up, years	Mean growth rate, mm/year^a^
Coady 1997 [2]	TAA > 35	1985–1996	16	ND	ND	Chronic dissection	CT, MRI, TTE	2.2	3.7 (1.3–6.1)
			63	ND	ND	No dissection			0.9 (0.3–1.5)
			54	ND	ND	Ascending aorta			1.0 (0.4–1.6)
			25	ND	ND	Descending aorta			2.0 (0.9–5.1)
Davies 2002 [3]	TAA > 35	1985–2000	129	ND	ND	Chronic dissection	CT, MRI, TTE	1.6	1.4
			203	ND	ND	No dissection			0.9
			332^b^	ND	ND	Ascending aorta			0.7
				ND	ND	Descending aorta			1.9
La Canna 2006 [18]	aTAA 40–60	1992–2003	27	49	96	BAV	TEE, TTE	3.0	0.81 ± 1.1, ns
			86	61	80	TAV		3.3	0.75 ± 1.1, ns
Davies 2007 [11]	aTAA > 35	1985–2005	70	49	74	BAV	CT, MRI, TTE	3.6	1.9 (1.3–2.5)*
			451	64	63	TAV		3.5	1.3 (1.1–1.5)*
			521	ND	ND	Mixed AV/		ND	1.4 (1.2–1.6)
Etz 2010 [19]	BAV-TAA	1988–2008	90	ND	ND	BAV	CT, partly digitised	4.2	0.77
Angeloni 2015 [20]	aTAA	2005–2011	329^c^	68	62	Statin	TTE, added CT	3	0.95 ± 0.44***
			329^c^	64	62	No statin		3	1.27 ± 0.56***

*aTAA* ascending thoracic aortic aneurysm, *AR* aortic regurgitation, *AS* aortic stenosis, *AV* aortic valve, *BAV* bicuspid aortic valve, *CT* computed tomography, *MRI* magnetic resonance imaging, *ND* no data, *TAA* thoracic aortic aneurysm, *TAV* tricuspid aortic valve, *TEE* transoesophageal echocardiography, *TTE* transthoracic echocardiography

^a^95% confidence interval is given in parentheses

^b^Not specified

^c^After propensity matching

*ns* not significant; *p* > 0.05; *p ≤ 0.05; ***p < 0.001

**Table 2:** Studies published since 2016

Study	Objective, mm	Period	Participants	Mean age, years	Male, %	AV type	Imaging	Mean FU, years	Mean growth rate, mm/year^a^
Gagné-Loranger 2016 [21]	aTAA 40–50	2001–2015	251	65	71	22% BAV	CT-ce axial	4.3	0.42 ± 0.82
Kim 2016 [22]^b^	aTAA 40–55	2001–2014	270	–	–	BAV	TTE	3.9	0.20(0.12–0.28)***
			1144	–	–	TAV			0.08(0.04–0.12)***
			1414	66	80	Mixed			0.10(0.06–0.14)
Park 2017 [16]	aTAA ≥ 40	2003–2014	509	67	56	5% BAV	CT-ce axial	4.3	0.3 ± 0.5
Trinh 2017 [23]	BAV-TAA	2009–2014	20	45	80	BAV	CE-MRA	2.6	0.5 ± 0.8
Agnese 2019 [24]	aTAA > 2.1 cm/m^2^	2000–2017	41	57	83	BAV	TTE	4	BAV vs TAV ns
			165	69	84	TAV			
			206	–	–	Mixed	TTE	4	0.46 ± 0.04
Rooprai 2019 [29]	BAV-TAA	2014–2018	29	57	66	BAV	TTE, CT, MRI	2.9	0.75 ± 0.81
Adriaans 2021 [17]	aTAA > 40	1999–2019	57	52	67	BAV	CT, MRI, TTE	6.7	0.2 ± 0.3 ns
			275	66	76	TAV			0.2 ± 0.4 ns
			332	64	75	Mixed			0.2 ± 0.4
Van Hout 2021 [28]	TAA	ND	31	55	74	TAV	MRI	8.0	0.39 ± 0.33
Korpela 2022 [25]	aTAA > 40	2017–2020	30	66	80	TAV	4D flow MRI	1.0	0.9
Weininger 2022 [26]	aTAA > 40	2013–2016	207	74	84	Mixed	CT-do	2.7	0.13 (median, IQR 0.24–0.49)
Gulati 2022 [27]	aTAA > 40	–	67	68	100	7% BAV	CT-do, 91% CTA	4.1	0.11 ± 0.31
Kauhanen 2022 [30]	aTAA > 40	2013–2018	143	64	80	12% BAV	CTA, ECG-gt	3.4	0.2 ± 0.5
Hiratzka 2023 [31]	aTAA 40–49	2015–2020	197	62	69	5% BAV	CT/MRI-oe	2.9	0.022 (−0.01 to +0.04)
Pace 2023 [32]	aTAA > 39	2002–2022	432	ND	ND	ND	CT, 71% CTA, 44% ECG-gt	4^c^	0.1 (0.1–0.2)

^a^ 95% confidence interval is given in parentheses

^b^ With additional data requested from authors

^c^ Median follow-up

*ns* not significant; *p* > 0.05; *p ≤ 0.05; ***p < 0.001

*aTAA* ascending thoracic aortic aneurysm, *AV* aortic valve, *BAV* bicuspid aortic valve, *CT* computed tomography, *CTA* computed tomography angiography, *CT-ce* contrast enhanced, *CT-do* double oblique, *CT-oe* outer edge, *ECG-gt* ECG gated, *FU* follow-up, *IQR* interquartile range, *MRA* magnetic resonance angiography, *MRI* magnetic resonance imaging, *ND* no data, *TAA* thoracic aortic aneurysm, *TAV* tricuspid aortic valve, *TTE* transthoracic echocardiography

**Fig. 4:**
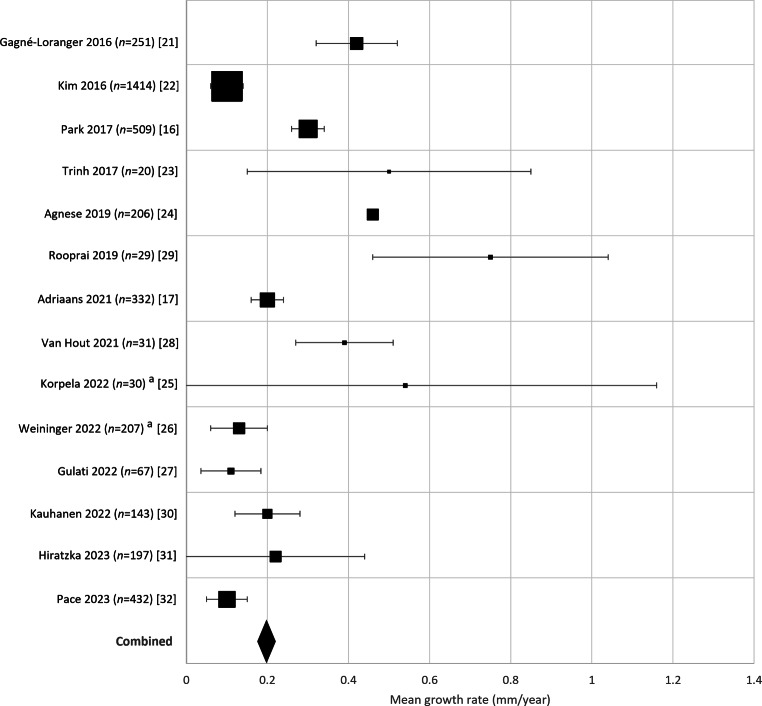
Growth rates from 14 studies since 2016 in patients with tricuspid aortic valve and/or bicuspid aortic valve (not specified in Pace et al. 2023 [32]). *Square size* indicates sample size, *error bars* indicate 95% confidence interval (95% CI). *Diamond* indicates the weighted mean growth rate of the combined studies (0.20 mm/year, SD 0.73) and its 95% CI (0.18–0.22); *n* = 3096. ^a^ Mean and standard deviation estimated from the median and interquartile range as reported by the authors [25, 26]

From Fig. 2 an apparent decrease in mean annual growth rates in studies published before 2016 and from 2016 onwards can be appreciated. Meta-regression analysis showed that the mean annual growth rate of the six studies published before 2016 was significantly higher than that of the 14 studies published in 2016 or later (*p* < 0.001). The pooled mean growth rate of the 14 studies published since 2016 was 0.20 mm/year (95% CI 0.18–0.22), irrespective of aortic valve type (Fig. 4).

### Growth rates in TAV versus BAV

Of the 14 studies published in 2016 or later, two included only patients with TAV [25, 28], two included only patients with BAV [23, 29], and three included both patients with TAV and BAV in subgroups [17, 22, 24]. In the latter three studies, aortic growth rates were directly compared between valve types. One study found lower annual growth rates in patients with TAV than in those with BAV (0.08 mm/year (95% CI 0.04–0.12) vs 0.20 mm/year (95% CI 0.12–0.28); *p* < 0.001) [22]. This, however, was not confirmed in the other two smaller studies [17, 24].

**Fig. 5:**
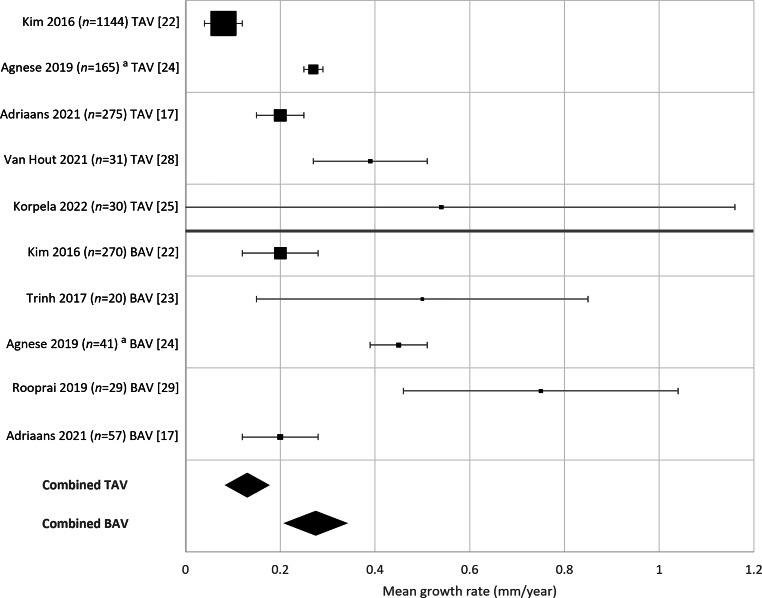
Differences in growth rates between tricuspid aortic valve (*TAV*) and bicuspid aortic valve (*BAV*) in seven ascending thoracic aortic aneurysm studies since 2016. *Square size* indicates sample size, *error bars* indicate 95% confidence interval (95% CI). *Diamonds* indicate the weighted mean growth rate of the combined studies for TAV (0.13 mm/year, SD 0.65, *n* = 1645 patients) and BAV (0.28 mm/year, SD 0.64, *n* = 417) and their 95% CI. ^a^Mean annual growth rates were not reported separately for TAV and BAV subgroups [24]. The mean and standard deviation for the respective subgroups were estimated from the annual results as reported in the paper (i.e. 0.27 mm/year (SD 0.12) for TAV and 0.45 mm/year (SD 0.20) for BAV)

Combining all seven studies, the weighted mean annual growth rate was lower in TAV than in BAV patients but did not reach statistical significance (0.13 (95% CI 0.10‑0.17) mm/year vs 0.28 (95% CI 0.22–0.34) mm/year; *p* = 0.288) (Fig. 5).

### Growth rates in relation to aneurysm size and follow-up duration

Study results concerning the association between initial aneurysm size and growth rate have been conflicting. Some have suggested increased growth rates in larger aneurysms (0.8 mm/year in aneurysms up to 40 mm vs 1.5 mm/year in aneurysms up to 80 mm) [2], while others have found increased growth rates only in aneurysms ≥ 50 mm [16, 22], or ≥ 55 mm ([32]; Tab. 3). One study failed to observe differences in growth rate between aneurysms ≤ 45 mm and > 45 mm [27], while one study even observed an inverse relation between annual growth rate and aneurysm size (Tab. 3; [21]). Again, another study reported increased growth rates only during the initial 2 years of follow-up, steadily decreasing, with the aortic diameter ultimately reaching a plateau [24].

**Table 3:** Mean ascending thoracic aortic aneurysm (*aTAA*) growth rates related to the diameter of the aneurysm

Study	Objective, mm	Participants	Mean follow-up, years	Baseline diameter, mm	Mean growth rate, mm/year^a^
Coady 1997 [2]	aTAA, > 35	54	2.2	40	0.8 (0.3–1.2)
				50	1.0 (0.4–1.5)
				60	1.1 (0.5–1.8)
				70	1.3 (0.6–2.1)
				80	1.5 (0.6–2.4)
Gagné-Loranger 2016 [21]	aTAA, 40–50	65	4.3	40–44	0.55 ± 0.77
		186		45–50	0.38 ± 0.84 ns
Park 2017 [16]	aTAA, ≥ 40	321	4.3	40–44	0.3 ± 0.5
		142		45–49	0.3 ± 0.5 ns
		46		≥ 50	0.7 ± 0.9**
Gulati 2022 [27]	aTAA, ≥ 40	46	4.1	≤ 45	0.09 ± 0.32
		21		> 45	0.14 ± 0.31 ns
Pace 2023 [32]	aTAA, > 39	43	1.0^b^	< 40	0.2 (0.1–0.3)
		200	0.9^b^	40–44	0.1 (0.1–0.2)
		130	0.7^b^	45–49	0.1 (0.03–0.1)
		41	0.8^b^	50–54	0.2 (0.1–0.3)
		12	0.9^b^	≥ 55	1.9 (1.2–2.5)*

^a^ 95% confidence interval is given in parentheses

^b^ Median follow-up

*ns* not significant; *p* > 0.05; *p < 0.05; **p < 0.01

Interestingly, several studies found that in 38–41% of patients after an initial increment, aTAA growth stabilised during follow-up [17, 26, 27]. More rapid aTAA growth was observed in a minority of patients (4–8%) [16, 17, 21, 27]. Because only a few patients were identified in a limited number of studies, each using different definitions for rapid growth, no meaningful conclusions on clinical risk features could be drawn.

### Certainty assessment

The results of our systematic review may be classified as Step 1 (Level 1) evidence for a prognostic research question according to the OCEBM classification.

## Discussion

This systematic literature review assessed growth rates in aTAA patients in relation to year of publication, allowing comparison between early landmark studies that form the basis for prevailing guidelines and more recent studies. A significant difference in the pooled mean growth rate was found between early and recent publications (1.17 (95% CI 1.01–1.33) vs 0.35 (95% CI 0.33–0.37) mm/year; *p* = 0.008). Taking only the studies published in 2016 and later into consideration, the pooled mean growth rate was even lower (0.20 mm/year (95% CI 0.18–0.22)). There was no statistically significant difference in mean annual aTAA growth rate between patients with TAV and BAV.

### *Potential explanations for lower mean aTAA growth rates in recent studies*

#### Differences in patient selection and follow-up duration

The early landmark studies included patients with more extreme pathology, such as initial aneurysm diameters up to 80 mm and patients with MFS and/or chronic dissections [2, 3]. More recent studies generally did not include patients with diameters > 55 mm, probably because—at that time—these patients qualified for pre-emptive surgery. Also, patients with connective tissue diseases or prior aortic diseases were generally excluded in recent publications.

Difference in follow-up duration is another factor that may explain the observed differences in growth rates between early and recent studies. In studies with short follow-up (as was the case in the three early studies, see Tab. 1) small diameter changes or measurement inaccuracies might have translated into disproportionately higher growth rates. More recent studies had substantially longer follow-up durations, during which such changes or inaccuracies are likely to become less apparent (Tab. 2).

#### Differences in imaging techniques and measurement methods

Different imaging modalities such as echocardiography, magnetic resonance imaging and computed tomography angiography (CT) can be used to measure aortic diameters, each technique having its specific advantages and disadvantages [33, 34]. The quality of aortic image acquisition and post-processing has obviously improved over time. The ascending aortic diameter has long been measured manually on axial CT images, which is now acknowledged to overestimate aortic diameters [8] compared to double oblique reconstructions and centreline measurements. The latter has now become standard and routine clinical practice. Finally, the advent of ECG gating has led to a better delineation of the aortic wall, hereby reducing measurement inaccuracies [8].

#### Differences in treatment

Early studies on aTAA growth were mainly focused on the *pure* ‘natural history’ of unselected patients. In later studies, selection of patients was more strict, standardised follow-up imaging was more often used, and anti-hypertensive medication was administered. For instance, in the study by La Canna et al., beta blockers and angiotensin-converting enzyme inhibitors were used in only 35 and 30% of patients [18], while the proportion was 77 and 80%, respectively, in the more recent study by Angeloni et al. [20]. The increasing awareness of the importance of medical therapy may have contributed to the observed lower aTAA growth rates in recent decades [21, 22, 24].

### Rapid aTAA growth

It is important to identify individuals with rapid aTAA growth, defined as a diameter increase of ≥ 3 mm/year in two consecutive years or ≥ 5 mm in one year. The 2022 ACC/AHA guidelines still recommend pre-emptive surgery in these patients [35]. Although we could not retrieve the prevalence of patients with rapid aTAA growth, a recent study found a growth rate > 3 mm/year in only 4.7% out of 444 patients [36]. Therefore, most patients with non-syndromic (sporadic) aTAAs have lower growth rates, suggesting that surveillance intervals may generally be prolonged.

### Study limitations and strengths

An important limitation of this review is the heterogeneity in study designs and the use of different imaging techniques and measurement methods in the studies included. Nevertheless, and despite guideline recommendations, the use of different imaging techniques and non-standardised follow-up intervals even in a single patient is still common in daily practice, especially during long-term follow-up.

Patient heterogeneity cannot be avoided in this review. Non-syndromic aTAAs will mostly be sporadic or BAV aortopathy, but we cannot exclude the fact that (some of) the selected studies may have included some patients with undiagnosed connective tissue disease.

### Clinical implications of lower mean aTAA growth rates

The much lower than previously assumed growth rate of non-syndromic (sporadic) aTAAs suggests that surveillance and imaging intervals may be prolonged in most patients. Implementation of adjusted imaging intervals might be a topic for future guideline revisions.

## Conclusion

The weighted mean growth rate of aTAAs in recent studies was much lower than observed in three early landmark studies (0.35 mm/year vs 1.17 mm/year). An even lower weighted mean growth rate was derived from studies published since 2016 (0.20 mm/year). Annual growth rate of aTAAs was not different between patients with BAV and TAV. Our findings suggest that the guideline-recommended annual interval for imaging of aortic diameters might be prolonged. Further research is needed to determine in which patients a less frequent follow-up approach is safe.

## Supplementary Information

Complete literature search terms (search date 28/11/2023)
