# Supplementary material for: Growth rates in non-syndromic aneurysms of the ascending aorta: a systematic review
Source: Neth Heart J. 2024 Nov 19;32(12):433–41. doi: 10.1007/s12471-024-01911-6 (PMC11584832; doi:10.1007/s12471-024-01911-6)
Supplement: Supplementary file 1 — Complete literature search terms (search date 28/11/2023) [file 12471_2024_1911_MOESM1_ESM.docx]

**Growth rates in non-syndromic aneurysms of the ascending aorta: a literature review**

L. Cozijnsen^1*^, BP. Adriaans^2,3,4^, TR. Schermer^5^, MGroenink^6^, Schalla^2,3,4^, SCAM. Bekkers^7^

^1^Department of Cardiology, Gelre Hospital, Apeldoorn, The Netherlands

^2^Cardiovascular Research Institute Maastricht (CARIM), Maastricht University, Maastricht, The Netherlands

^3^Department of Radiology and Nuclear Medicine, Maastricht University Medical Centre, Maastricht, The Netherlands

^4^Department of Cardiology, Maastricht University Medical Centre, Maastricht, The Netherlands.

^5^Department of Clinical Epidemiology and Statistics, Gelre Hospital, Apeldoorn, The Netherlands

^6^Department of Cardiology, Amsterdam University Medical Centre, Amsterdam, The Netherlands.

^7^Department of Cardiology, Bernhoven Hospital, Uden, The Netherlands

**Electronic Supplementary Material** Complete literature search terms (search date 28/11/2023)

**("****Aorta, Thoracic"[Mesh] OR Thoracic Aorta[tiab] OR TAA[tiab] OR "Aortic Aneurysm, Thoracic"[Mesh] OR "Thoracic Aortic Aneurysm"[tiab] OR "Thoracic Aortic Aneurysms"[tiab] OR "Dissecting Aneurysm"[tiab] OR "Dissecting Aneurysms"[tiab] OR "Aortic Dissections"[tiab] OR "Aortic Dissection"[tiab] OR "Aortic Dissection"[Mesh] OR aortic root[tiab] OR ascending aortic[tiab] OR thoracic aortic[tiab] OR ascending aorta[tiab])**

**AND**

**(Growth rate[tiab] OR dilatation rate[tiab] OR progression rate[tiab] OR (natural history[tiab] AND aneurysm*[tiab]) OR diameter progression[tiab] OR millimeter progression[tiab] OR moderately dilated[tiab])**
